# First record of *Scincellafansipanensis* Okabe, Motokawa, Koizumi, Nguyen, Nguyen & Bui, 2024 (Squamata, Scincidae) from China

**DOI:** 10.3897/BDJ.13.e161782

**Published:** 2025-08-12

**Authors:** Yuhao Xu, Chencan Liao, Nikolay Poyarkov, Shiyang Weng, Jundong Deng, Andrei Bragin, Tierui Zhang, Nguyen Van Tan, Lifang Peng

**Affiliations:** 1 State Key Laboratory of Plateau Ecology and Agriculture, Qinghai University, Xining, China State Key Laboratory of Plateau Ecology and Agriculture, Qinghai University Xining China; 2 Ailaoshan Subtropical Forest Ecosystem Observation and Research Station of Yunnan Province, Puer, China Ailaoshan Subtropical Forest Ecosystem Observation and Research Station of Yunnan Province Puer China; 3 Xi Shuang Ban Na Tropical Botanical Garden, Chinese Academy of Sciences, Dai Autonomous Prefecture of Xishuangbanna, China Xi Shuang Ban Na Tropical Botanical Garden, Chinese Academy of Sciences Dai Autonomous Prefecture of Xishuangbanna China; 4 Department of Vertebrate Zoology, Lomonosov Moscow State University, Moscow, Russia Department of Vertebrate Zoology, Lomonosov Moscow State University Moscow Russia; 5 Tibet Plateau Institute of Biology, Lhasa, China Tibet Plateau Institute of Biology Lhasa China; 6 Joint Vietnam - Russia Tropical Science and Technology Research Centre, Hanoi, Vietnam Joint Vietnam - Russia Tropical Science and Technology Research Centre Hanoi Vietnam; 7 Anhui Province Key Laboratory of the Conservation and Exploitation of Biological Resource, College of Life Sciences, Anhui Normal University, Wuhu, China Anhui Province Key Laboratory of the Conservation and Exploitation of Biological Resource, College of Life Sciences, Anhui Normal University Wuhu China; 8 The School of Medicine & Pharmacy, Duy Tan University, Da Nang, Vietnam The School of Medicine & Pharmacy, Duy Tan University Da Nang Vietnam; 9 Center for Entomology & Parasitology Research, Duy Tan University, Da Nang, Vietnam Center for Entomology & Parasitology Research, Duy Tan University Da Nang Vietnam

**Keywords:** *Scincellapotanini*–*S.monticola* complex, morphology, new record, phylogeny, *Scincella*, Ailao Mountain

## Abstract

**Background:**

The Fansipan ground skink, *Scincellafansipanensis* Okabe, Motokawa, Koizumi, Nguyen, Nguyen & Bui, was recently described from Fansipan Mountain, Lao Cai Province, northern Vietnam. Herein, we report the first national record of this species from China, based on four specimens collected from Ailao Mountain, Jingdong Yi Autonomous County, Yunnan Province.

**New information:**

Morphologically, the Chinese specimens are consistent with the type series, but differ slightly in possessing a higher number of ventral scales (including gulars) and relatively shorter fore-limbs. Phylogenetic analyses, based on *CO1* sequences, place the Chinese specimens within the *S.fansipanensis* clade; however, uncorrected *p*-distances of 6.5–7.3% between the Chinese and Vietnamese populations suggest moderate genetic divergence. This level of differentiation may reflect historical isolation by the Red River Basin's complex topography and the low dispersal ability of *Scincella* species. This record raises the number of the genus *Scincella* species known from China to 15.

## Introduction

The smooth skink genus *Scincella* Mittleman, 1950 is a widespread group of semi-fossorial lygosomine skinks, currently comprising 48 recognised species, distributed across North and Central America and extending through South, East and Southeast Asia (Ouboter 1986, Zhao et al. 1999) In Asia, the genus exhibits particularly high species richness in southern China and mainland Indochina, including Cambodia, Laos and Vietnam. Despite this diversity, the true species richness of *Scincella* is likely underestimated, as numerous cryptic lineages have been revealed through recent integrative taxonomic studies, yet remain undescribed (e.g. Neang et al. (2018), Nguyen et al. (2019), Nguyen et al. (2020), Koizumi et al. (2022), Jia et al. (2023), Jia et al. (2024), Okabe et al. (2024), Bragin et al. (2025), Nguyen et al. (2025)). These findings underscore the importance of continued field surveys and taxonomic research to clarify species boundaries and distribution patterns within the genus. In China, 14 species of the genus *Scincella* are currently recognised, including *S.barbouri* (Stejneger), *S.chengduensis* Jia, Ren, Jiang & Li, *S.doriae* (Boulenger), *S.formosensis* (van Denburgh), *S.huanrenensis* Zhao & Huang, *S.liangshanensis* Jia, Gao, Wu, Ren, Jiang & Wu, *S.modesta* (Günther), *S.monticola* (Schmidt), *S.potanini* (Günther), *S.przewalskii* (Bedriaga), *S.reevesii* (Gray), *S.schmidti* (Barbour), *S.tsinlingensis* (Hu & Zhao) and *S.wangyuezhaoi* Jia, Gao, Huang, Ren, Jiang & Li. This number is expected to increase with continued field surveys and taxonomic research (Zhao et al. 1999, Jia et al. 2023, Jia et al. 2024, Jia et al. 2025, Uetz et al. 2025). These species collectively reflect the high diversity of *Scincella* in southern and south-western China, particularly in mountainous and forested regions.

The Fansipan ground skink, *Scincellafansipanensis* Okabe, Motokawa, Koizumi, Nguyen, Nguyen & Bui, 2024, is assigned to the *S.potanini*-*S.monticola* species complex, based on molecular evidence (Bragin et al. 2025). It was originally described from three males, five females and two juveniles and is currently known only from its type locality, Mount Fansipan in Lao Cai Province, north-western Vietnam. Morphologically, the species is characterised by a medium body size, with a maximum snout-vent length of 59.0 mm and an axilla-groin length of 36.5 mm; 22–24 mid-body scale rows; 60–68 paravertebral scale rows; 58–64 ventral scale rows; prefrontals separated; 5–6 supraciliaries; 2–6 enlarged nuchals; 10–12 enlarged lamellae beneath toe IV; toes not in contact with fingers when limbs are adpressed; ear opening present, tympanum deeply recessed, without lobules; and dorsal surface scattered with irregular dark spots (Okabe et al. 2024).

During recent herpetological surveys in the Ailao Mountains, Yunnan Province, southwest China in 2025, four specimens of the genus *Scincella* were collected. Subsequent morphological and molecular analyses indicate that these specimens are referable to *S.fansipanensis*. We therefore report the first national record of this species in China, provide a detailed description of the Chinese specimens and offer an updated diagnosis of the species.

## Materials and methods

### Sampling

**Figure 1.**
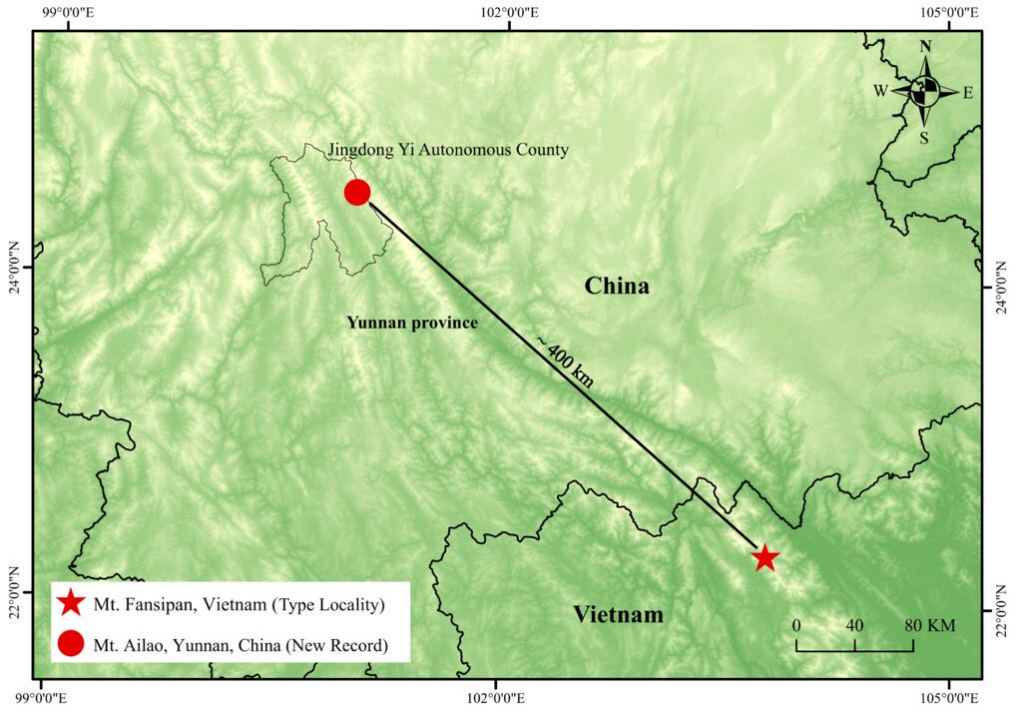
Map showing the new record in China (red circle) and the type locality of *Scincellafansipanensis* (red star) in Vietnam.

Field surveys were conducted in the Ailao Mountains, Jingdong Yi Autonomous County (Fig. 1), in April 2025 by Mr. Chenchan Liao from the Ailaoshan Subtropical Forest Ecosystem Observation and Research Station of Yunnan Province. During the survey, four *Scincella* specimens were collected. All individuals were euthanised using a lethal injection of 0.7% tricaine methanesulphonate (MS-222), then fixed and preserved in 75% ethanol for long-term storage. Fresh liver tissue was extracted, placed in 95% ethanol and subsequently stored at -20°C. All specimens were deposited in the Museum of Qinghai University, Qinghai, China (QHU). The related procedures complied with the Wildlife Protection Law of China and were approved by the Institutional Ethics Committee of Qinghai University (protocol number PJ202501-89).

### Molecular laboratory methods

Total genomic DNA was extracted from preserved liver tissue using the QIAamp DNA Mini Kit (QIAGEN, Changsheng Biotechnology Co. Ltd). Three mitochondrial gene fragments were amplified: 12S ribosomal RNA (*12S*) using the primer pair L1091-F (5'-AAACTGGGATTAGATACCCCACTAT-3') and H1478-R (5'-GAGGGTGACGGGCGGTGTGT-3') (Kocher et al. 1989); 16S ribosomal RNA (*16S*) using 16SL-F (5'-TGTTTACCAAAAACATAGCCTTTAGC-3') and 16SL-R (5'-TAGATAGAAACCGACCTGGATT-3') (Linkem et al. 2011); and cytochrome c oxidase subunit I (*CO1*) using L14724-F (5'-GACTTGAAAAACCACCGTTG-3') and EI700r-R (5'-GGGGTGAAAGGGGATTTT(AG)TC-3') (Nagy et al. 2012). Raw sequences were assembled using SeqMan in the DNASTAR software package (Burland 2000). The product was purified and sequenced by Shanghai Map Biotech Co., Ltd. All resulting sequences were deposited in GenBank under accession number (Table 1).

### Molecular phylogeny

The newly-obtained sequences, along with homologous sequences downloaded from GenBank, were aligned using MUSCLE (Edgar 2004) with default settings and then visually checked by eye for accuracy and trimmed to minimise missing characters in MEGA X (Kumar et al. 2018). *Sphenomorphuscryptotis* Darevsky, Orlov & Ho was selected as the outgroup, based on Jia et al. (2023). Uncorrected pairwise genetic distances (*p*-distances) between sequences were calculated in MEGA X (Kumar et al. 2018), based on *CO1* sequences of species within the *S.potanini*–*S.monticola* complex as defined by Bragin et al. (2025). Phylogenetic reconstruction using Maximum Likelihood (ML) was executed in IQ-TREE v.1.6.12 (Nguyen et al. 2015). To evaluate nodal support in the phylogenetic analyses, we employed both the Ultrafast Bootstrap Approximation (UFB) and the SH-like approximate likelihood ratio test (SH-aLRT). The UFB support values were calculated using 5000 bootstrap replicates, with values ≥ 95% regarded as strong support, following the approach of Hoang et al. (2018). In parallel, the SH-aLRT was performed with 1000 replicates and nodal support values ≥ 80% were considered well supported, in line with the criteria proposed by Stephane et al. (2010). The resulting phylogenetic tree was visualised in FigTree v.1.4.4 (Rambaut 2018).

### Morphological examination

Morphological data, including both meristic and morphometric characters, were described following the methodology of Bragin et al. (2025), with certain abbreviations revised according to the standards of Darko et al. (2022). Body measurements such as Snout-vent Length (**SVL**), Tail Length (**TAL**) and axilla-groin distance (**AGD**) were measured with a Deli digital calipers (No. 90150B) to the nearest 0.1 mm. All other measurements were taken using Mitutoyo digital calipers (CD-15AX) to the nearest 0.01 mm. The measurements taken include: **HL1** = head length (distance from the tip of the snout to the articulation of jaw); **HL2** = distance from the tip of the snout to the rear edge of parietal scale; **HW** = head width, the widest portion between the left and right articulations of jaw; **HH** = head height, the deepest portion from ventral to dorsal surface of head; **ED** = eye diameter, the maximum horizontal diameter of eye; **EN** = eye‑narial distance, measured from the anterior margin of the eye to the posterior margin of the nare; **PDD** = palpebral disc diameter, the maximum horizontal diameter of palpebral disc; **ESD** = snout length, measured from the tip of the snout to the anterior corner of eye; **EL** = ear opening diameter, maximum diameter of tympanum; **FLL** = fore-limb length, measured from anterior junction of fore-limb and body wall to tip of the finger IV; **F4L** = finger IV length, measured from the junction between the skin of third and fourth fingers to tip of finger IV; **HLL** = hind‑limb length, measured from anterior junction of hind-limb and body wall to tip of toe IV; **T4L** = toe IV length, measured from the junction between the skin of third and fourth toes to tip of toe IV; and **Limbs adpressed** = whether the fore-limbs and hind-limbs can make contact when the body is held straight and the limbs are adpressed.

Scalation features and their abbreviations are as follows: **SL** = supralabials; **IL** = infralabials; **FrN** = frontonasals; **SCI** = superciliaries; **SO** = supraoculars; **PF** = prefrontals; **FrP** = frontoparietals; **P** = parietals; **TEMP** = enlarged temporals; **Lor** = loreals; **NU** = nuchals, **CS** = chin‑shields; **GS** = gulars, number of scale rows counted from the first scale behind the chin shields to the mid-point of the line connecting the anterior edges of the fore-limb insertions; **MBSR** = mid-body scale rows, number of longitudinal scale rows measured around the widest point of mid-body; **PVSR** = paravertebral scale rows, the number of scale rows counted between parietals and the just posterior margin of hind-limbs; **DBR** = dorsal scale rows between dorsolateral stripes, the number of mid-body dorsal scale rows between dark dorsolateral stripes, including the half-scale on the scale where the upper border of the dark upper lateral zone lies; **SRB** = scale rows covered by dorsolateral stripes; **VS** = ventral scale rows, the number of scale rows counted between gulars and precloacals; **F4S** = number of enlarged, subdigital lamellae beneath finger IV; and **T4S** = number of enlarged, subdigital lamellae beneath toe IV.

The abbreviations of the diagnostic colouration characters in *Scincella* species are as follows: **UMLLS** = the upper margin of dorsolateral stripes wavy or relatively straight; and **VDM** = ventral dark markings, presence or absence of dark-coloured markings on the ventral surface.

Other abbreviations are as follows: **Is**: Island; **Mt.**: Mountain; **Mts.**: Mountains; **NP**: National Park; **NR**: Nature Reserve; **WS**: Wildlife Sanctuary; **a.s.l.**: above sea level.

## Taxon treatments

### Scincella fansipanensis

Okabe, Motokawa, Koizumi, Nguyen, Nguyen & Bui, 2024

F24ADD79-2271-5593-BD75-904868C285E5

#### Materials

- **Type status:** Other material. **Occurrence:** catalogNumber: QHU R2025004; sex: male; lifeStage: adult; disposition: in collection; occurrenceID: 4C7A8AF2-11A7-59C8-8FF7-44C583BB8B00; **Taxon:** scientificName: *Scincellafansipanensis*; order: Squamata; family: Scincidae; genus: Scincella; specificEpithet: *fansipanensis*; vernacularName: Fansipan ground skink; **Location:** country: China; stateProvince: Yunnan Province; county: Jingdong Yi Autonomous County; locality: Ailao Mountain; verbatimElevation: 2,480 m; verbatimCoordinates: 24.5419°N, 101.0294°E; **Event:** eventDate: April 2025; fieldNumber: LFR 2025038
- **Type status:** Other material. **Occurrence:** catalogNumber: QHU R2025005; sex: male; lifeStage: adult; disposition: in collection; occurrenceID: 58FD12E2-4399-5F52-80A8-4AED0A49D441; **Taxon:** scientificName: *Scincellafansipanensis*; order: Squamata; family: Scincidae; genus: Scincella; specificEpithet: *fansipanensis*; vernacularName: Fansipan ground skink; **Location:** country: China; stateProvince: Yunnan Province; county: Jingdong Yi Autonomous County; locality: Ailao Mountain; verbatimElevation: 2,480 m; verbatimCoordinates: 24.5419°N, 101.0294°E; **Event:** eventDate: April 2025; fieldNumber: LFR 2025039
- **Type status:** Other material. **Occurrence:** catalogNumber: QHU R2025006; lifeStage: juvenile; disposition: in collection; occurrenceID: 3F8FC54C-1F44-5822-98D2-F4CD5091A120; **Taxon:** scientificName: *Scincellafansipanensis*; order: Squamata; family: Scincidae; genus: Scincella; specificEpithet: *fansipanensis*; vernacularName: Fansipan ground Skink; **Location:** country: China; stateProvince: Yunnan Province; county: Jingdong Yi Autonomous County; locality: Ailao Mountain; verbatimElevation: 2,480 m; verbatimCoordinates: 24.5419°N, 101.0294°E; **Event:** eventDate: April 2025; fieldNumber: LFR 2025040
- **Type status:** Other material. **Occurrence:** catalogNumber: QHU R2025007; lifeStage: juvenile; disposition: in collection; occurrenceID: 4F3ED89C-2572-5C1F-90B2-CEAAE8F2F235; **Taxon:** scientificName: *Scincellafansipanensis*; order: Squamata; family: Scincidae; genus: Scincella; specificEpithet: *fansipanensis*; vernacularName: Fansipan ground skink; **Location:** country: China; stateProvince: Yunnan Province; county: Jingdong Yi Autonomous County; locality: Ailao Mountain; verbatimElevation: 2,480 m; verbatimCoordinates: 24.5419°N, 101.0294°E; **Event:** eventDate: April 2025; fieldNumber: LFR 2025041

#### Suggested common names

We suggest “黄连山滑蜥” (Huáng Lián Shān Huá Xī) as the Chinese common name and “Fansipanskiy malyi stsink” (Фансипанский малый сцинк) as the Russian common name.

#### Description of the specimens from China (n = 4)

The measurements and scalation features of the specimens are listed in Table 2. Size medium (SVL 30.1–51.5 mm, n = 4); tail relatively long, TAL/SVL ratio 1.78 in the only specimen with an original tail (QHU R2025006). Axilla-groin distance 16.5–30.1 mm, AGD/SVL ratio 0.55–0.59. Head elongated, indistinct from the neck (HL1 5.72–9.03 mm, HL2 5.18–7.82 mm, HW 4.00–5.29 mm, HH 2.83–4.69 mm). Snout short, obtuse, round anteriorly (ESD 1.57–2.39 mm, EN 1.06–1.93 mm). Eye large (ED 1.52–2.15 mm); lower eyelid with an undivided transparent palpebral disc (window) (PDD 0.58–0.89 mm). Ear oval; tympanum recessed and distinctly larger than the palpebral disc (EL 1.04–1.58 mm, EL/PDD ratio 1.73–1.93). Limbs relatively short, toes nearly touching fingers when limbs are adpressed (FLL 5.39–8.90 mm, HLL 7.02–12.02 mm, F4L 1.44–1.95 mm, T4L 2.02–3.57 mm, FLL/SVL ratio 0.16–0.18, HLL/SVL ratio 0.22–0.26). Digits moderately long and slender, each ending in a clearly visible, slightly curved claw.

##### Head scalation

Head scales smooth. Rostral convex, wider than high, distinctly visible from above, in contact with the first supralabials, nasals and frontonasal; supranasals absent; frontonasal single, approximately boat-shaped, with a width about twice its height, in contact with the rostral, nasals, anterior loreals, prefrontals and frontal; prefrontals two, not in contact with each other, separated medially by the frontal; frontal slender, longer than wide, diamond-shaped, in contact laterally with the first superciliary and the first and second supraoculars; a pair of frontoparietals in contact anteriorly, bordered by the frontal, second to fourth supraoculars, interparietal and parietals; interparietal diamond-shaped, width less than height; parietals large, in contact posteriorly, posterolateral margins bordered by the upper secondary temporals and enlarged nuchals; enlarged nuchals 2–4.

Nostril oval, situated centrally within the nasal; nasal entire, diamond-shaped, with width approximately equal to height, in contact with the rostral, frontonasal, first loreal and the first supralabial; loreals two, subequal in size; supraoculars 4/4, first contacting the frontal, second the largest, in contact with both the frontal and frontoparietals, third and fourth contacting the frontoparietals; superciliaries six (rarely five), with the first being the largest; palpebral disc bordered by a series of small scales; temporals arranged 1+2, anterior temporal subrectangular, upper secondary temporal the largest, lower secondary temporal smaller and broadly contacting the upper; supralabials 7/7, first the smallest, fifth positioned below the eye, sixth the largest.

**Figure 2.**
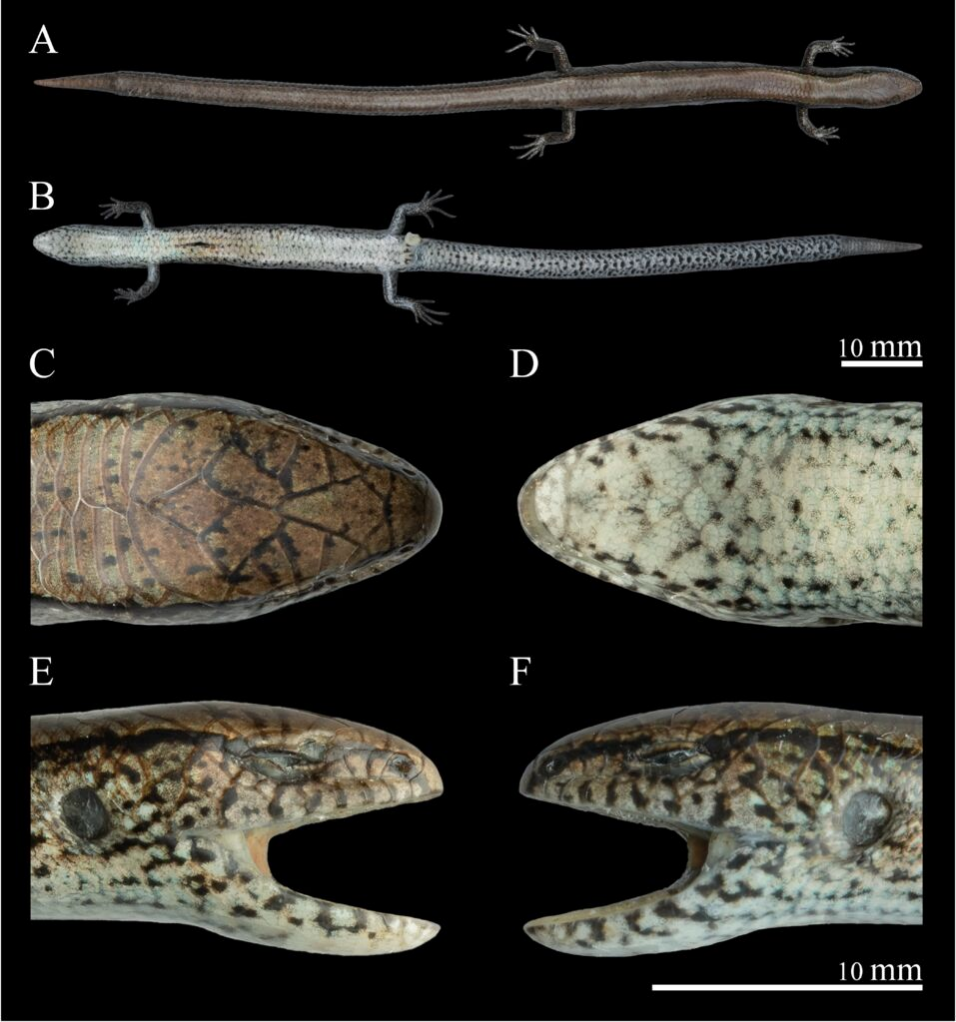
*Scincellafansipanensis* (QHU R2025004, adult male) from Yunnan, China, after one month in ethanol preservation - dorsal (A) and ventral view (B) of the whole body; dorsal (C), ventral (D), right (E) and left (F) view of the head. Photographs by Y.H. Xu. Scale bars: 10 mm.

Mental wider than long, round anteriorly, in contact with the first infralabials and the postmental; postmental large, subpentagonal, in contact the mental, the first two infralabials on each side and the first pair of chin shields; infralabials 6/6, first the smallest, fifth and sixth the largest; three pairs of chin shields, the first pair in medial contact, the second pair separated by one gular scale and the third pair separated by three gulars scales; gulars 19–22 (Fig. 2).

##### Body scalation

**Figure 3.**
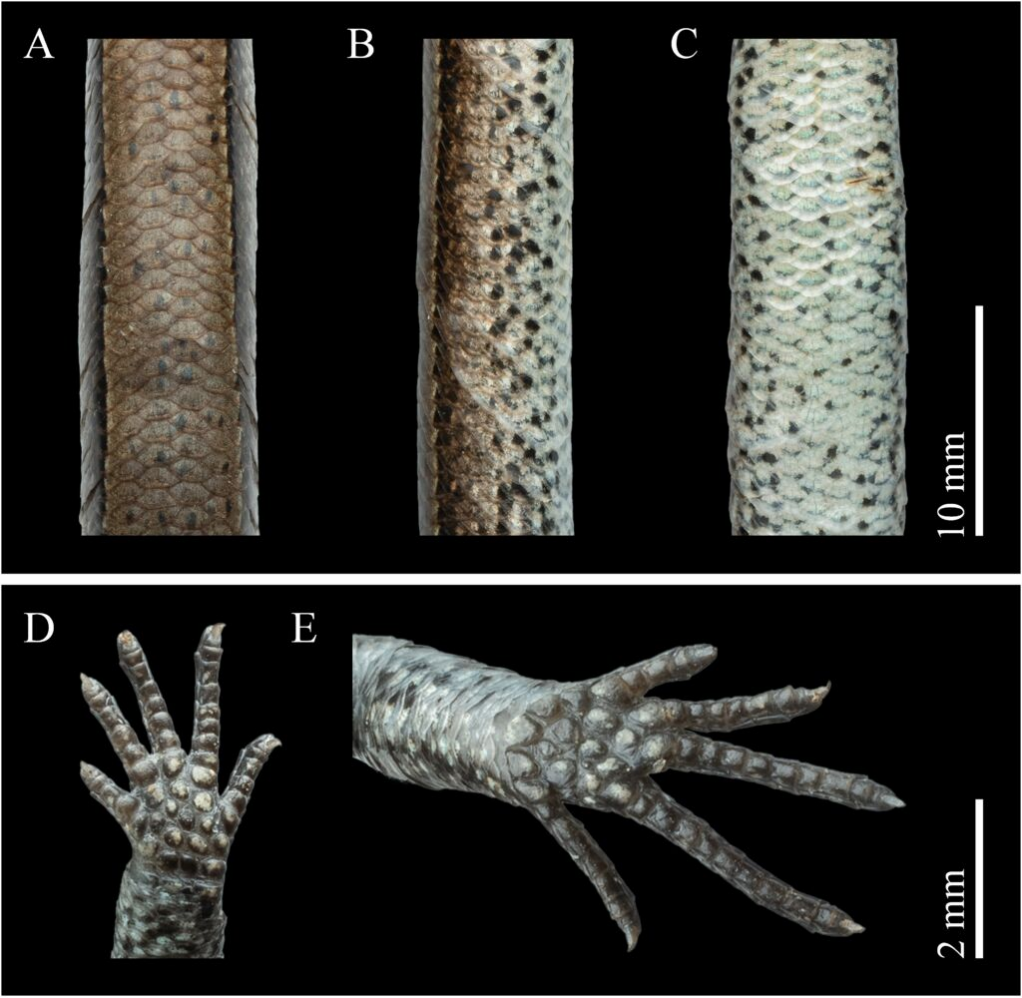
Dorsal (A), lateral (B) and ventral (C) views of the body; (D) ventral view of the hand; (E) ventral view of the foot of *Scincellafansipanensis* (QHU R2025004) from Yunnan, China, after one month in ethanol preservation. Photographs by Yuhao Xu. Scale bars in A–C: 10 mm, and D–E: 2 mm.

Body scales smooth, 22–24 rows around mid-body; distinctly larger dorsally than laterally and slightly larger than ventrals; paravertebral scale series composed of 64–71 scales; dorsal scales between dorsolateral stripes arranged as 1/2+4+1/2. Ventral scales slightly enlarged medially, decreasing in size towards the flanks, ventral scale rows (excluding gulars) 48–52, GS+VS 70–74; medial pair of precloacal scales enlarged, with the left overlapping the right. Tail complete; tail scales imbricate and generally uniform in shape, except for the markedly widened subcaudals. Limbs pentadactyl; dorsal surface of fingers and toes covered with two interdigitating scale rows; subdigital lamellae 7–8 beneath finger IV and 10–12 beneath toe IV (Fig. 3).

##### Colouration in life

The dorsal surface of the head is olive-brown, scattered with small, irregular dark spots; the head scales are margined with dark brown. Upper lateral margins of the head also olive-brown, gradually fading to light brown towards the lower sides and bearing slightly larger, irregular dark spots. Ventral surface of the head is creamy-white, scattered with irregular dark blotches.

**Figure 4.**
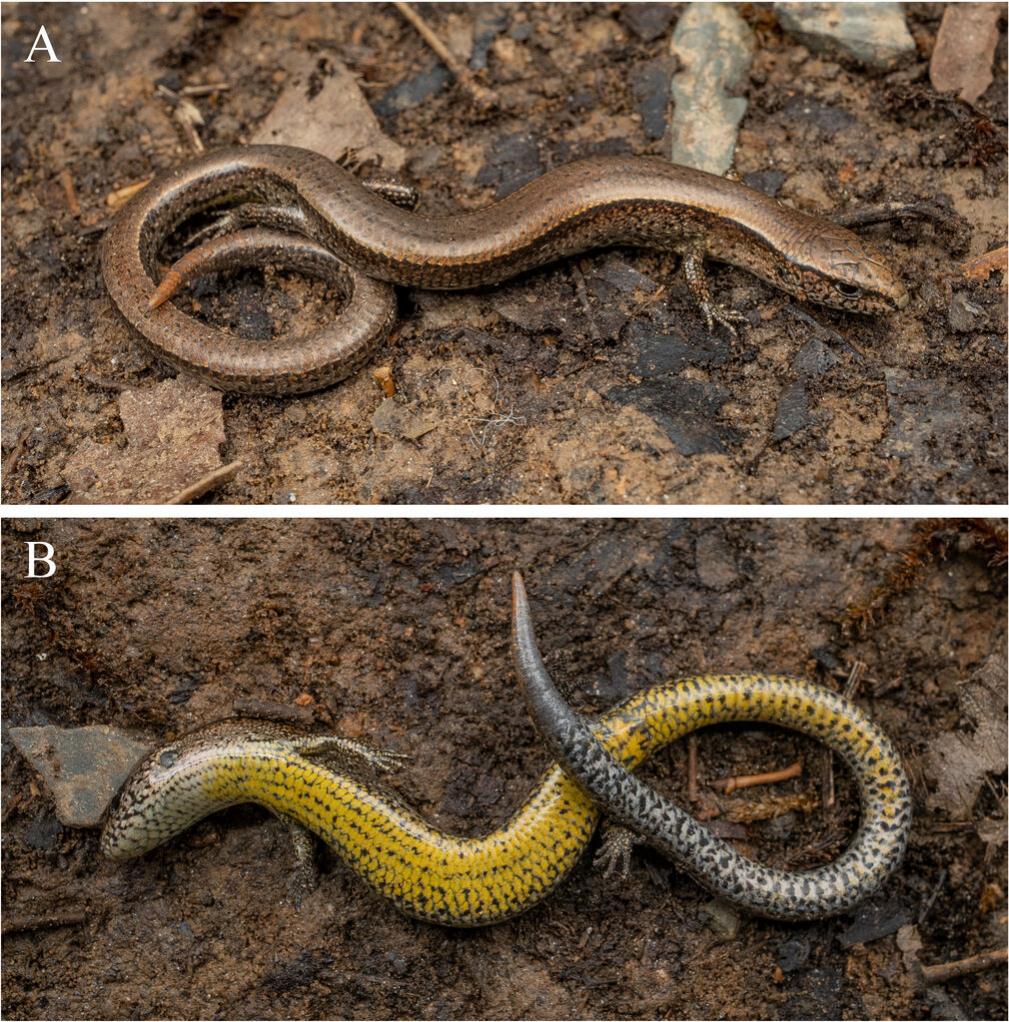
*Scincellafansipanensis* (QHU R2025004, adult male) from Yunnan, China in life - dorsal (A) and ventral (B) views. Photographs by Y.H. Xu.

The dorsal surface of the body and tail is olive-brown, scattered with small, irregular dark spots. Distinct dark dorsolateral stripes with relatively straight upper edges extend from the tip of the snout, are interrupted at the eye, resume posterior to the eye and continue along the flanks above the fore-limbs and hind-limbs, reaching the tail; and these stripes cover approximately 1.5–2 scale rows on the trunk. Ground colour of the upper flanks brown, gradually transitioning ventrally to a light brownish-yellow and scattered with black spots. Ventral surface of the trunk yellow, sometimes with dark spots, these spots being more concentrated laterally and along the junction between the flanks and ventral surface, but becoming sparse or absent toward the mid-line of the belly. Anterior portion the ventral tail pale yellow, gradually transitioning to grey or grey-white posteriorly, with scattered black spots (Fig. 4).

##### Colouration in preservation

**Figure 5.**
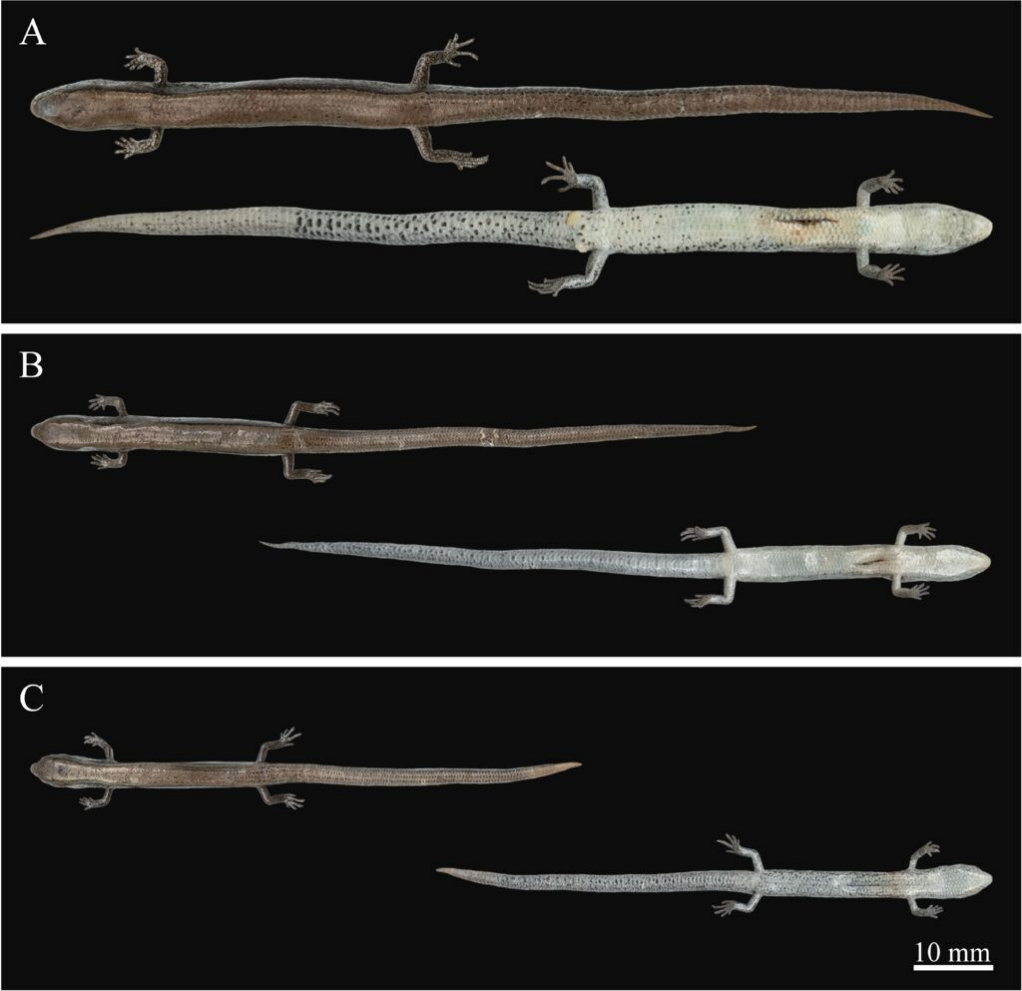
Colouration of additional specimens of *Scincellafansipanensis* from Yunnan, China, after one month in ethanol preservation. **A** QHU R2025005, adult male; **B** QHU R2025006, juvenile; **C** QHU R2025007, juvenile. Photographs by Y.H. Xu. Scale bars: 10 mm.

After one month in ethanol, the colouration remains generally similar to that in life, except that the ground colour of the lower flanks and ventral surface of the trunk has turned creamy-white and the ventral surface of the tail has become completely greyish-white (Figs 2, 5).

#### Variation

Compared to the type series from Vietnam, the Chinese specimens are nearly identical in colouration, except that most individuals possess dark spots on the ventral surface of the trunk. In terms of morphometric and scalation characters, the two populations are also highly similar, except that the Chinese population exhibits a higher number of ventral and gular scales combined (VS+GS 70–74 vs. 58–64) and relatively shorter fore-limbs (FLL/SVL ratio 0.16–0.18 vs. 0.19–0.22).

#### Revised diagnosis

(1) size medium within genus *Scincella* (SVL up to 59.0 mm and AGD up to 36.5 mm); (2) mid-body scale rows 22–24; (3) paravertebral scale rows 60–71; (4) VS+GS 58–74; (5) prefrontals separated from each other; (6) supraciliaries 5–6; (7) supralabials 6–7; (8) infralabials 6; (9) nuchals 2–6; (10) ear opening present, tympanum deeply sunk, without lobules; (11) 7–9 enlarged lamellae beneath finger IV and 10–12 beneath toe IV; (12) toes not in contact with fingers when limbs adpressed; (13) the dark dorsolateral stripes with relatively straight upper edge, covering about 1.5–2.5 scale rows on the trunk, with four scale rows in between on the dorsum; (14) dorsal surface of body olive-brown, scattered with irregularly-shaped dark sports (data from Okabe et al. (2024) and this study).

#### Distribution and natural history notes

**Figure 6.**
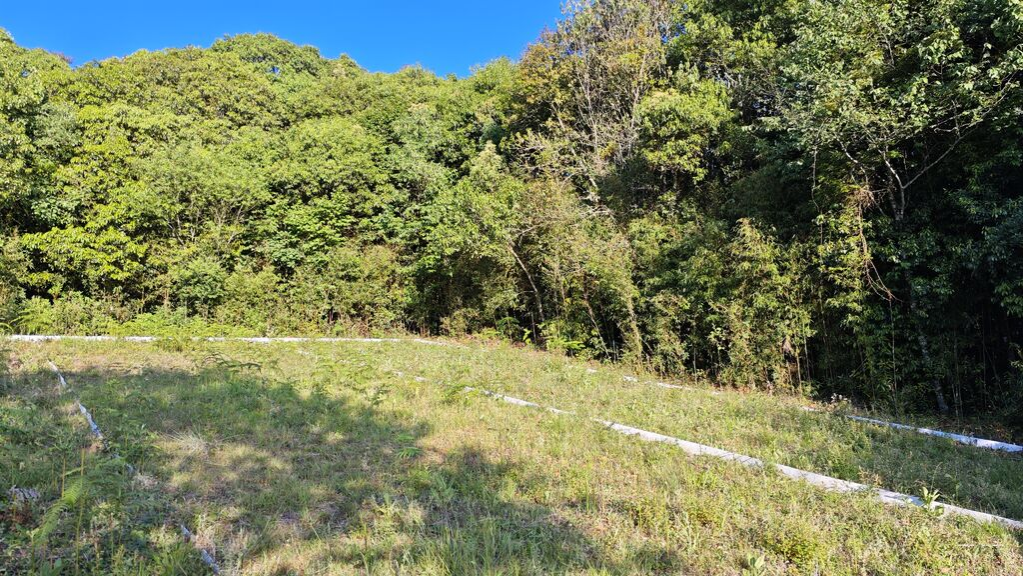
Habitat of *Scincellafansipanensis* in Mt. Ailao , Yunnan, China. Photograph by C.C. Liao.

*Scincellafansipanensis* is currently known from the type locality in Hoang Lien Son Range, Lao Cai Province, north-western Vietnam and from Jingdong Yi Autonomous County, Pu’er City, central Yunnan Province, China (Fig. 1). All specimens from Mt. Ailao were collected in April 2025 at an elevation of 2,480 m a.s.l., in grassy areas along the forest edge (Fig. 6). The surrounding habitat consists of well-preserved broadleaf forest. During the survey, daytime temperatures averaged approximately 25°C, dropping to about 18°C at night.

Field observations suggest that these skinks prefer areas with lower canopy cover, such as forest gaps or ecotones between forest and human settlements. These habitats areas are typically more open, receive greater sunlight during the day and support dense herbaceous vegetation that offers ample shelter. In contrast, individuals were rarely encountered in densely shaded forest interiors where minimal light reaches the ground.

## Analysis

**Figure 7.**
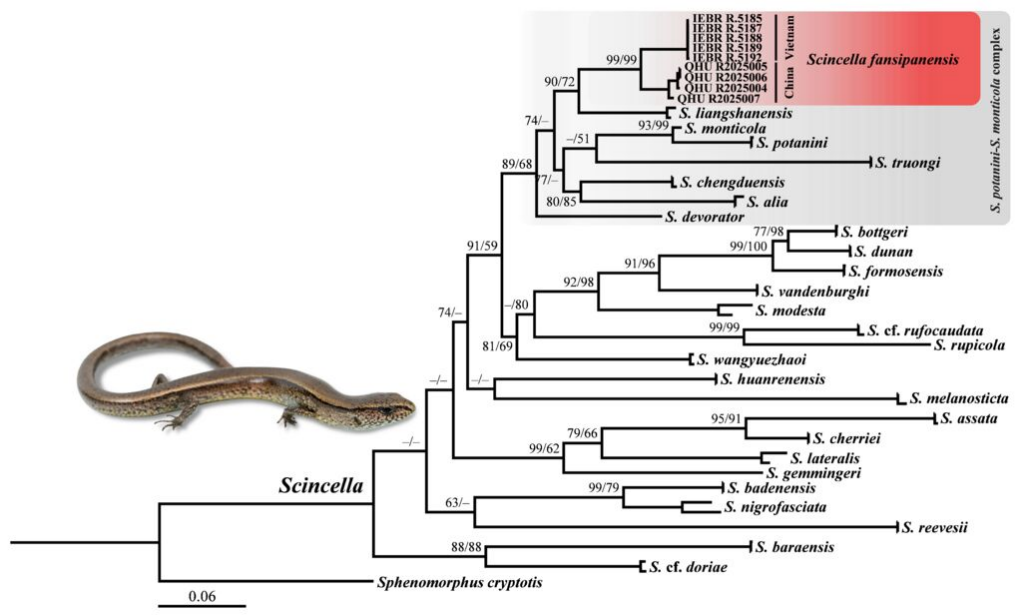
Phylogenetic topology of the genus *Scincella* inferred from three mitochondrial (*12S*/*16S*/*CO1*) fragments. The nodes supporting values on branches are presented with the SH-like approximate likelihood ratio test (SH)/Ultrafast Bootstrap Approximation (UFB); the ones lower than 50 are displayed as “–”. Photograph on thumbnails by Y.H. Xu.

The lengths of the *12S*, *16S* and *CO1* fragments in the final alignment comprised 384 base pairs (bp), 542 bp and 659 bp, respectively. The ML analysis (Fig. 7) yielded a topology broadly congruent with previous studies, based on similar datasets (Jia et al. 2023, Jia et al. 2024, Bragin et al. 2025, Jia et al. 2025). A monophyletic clade was recovered comprising *Scincellaalia*, *S.chengduensis*, *S.liangshanensis*, *S.fansipanensis*, *S.potanini*, *S.monticola*, *S.truongi* Pham, Ziegler, Pham, Hoang, Ngo & Le and *S.devorator* (Darevsky, Orlov & Ho), although with relatively low support (SH 89/UFB 68). The specimens collected from Ailao Mt., Yunnan Province, China clustered with the type series of *S.fansipanensis*, including the holotype, with strong support (SH 99/UFB 99). A moderate level of genetic divergence was observed between the Chinese and Vietnamese populations, with uncorrected pairwise *p*-distances, based on the *CO1* gene, ranging from 6.5% to 7.3% (Table 3). However, these values remain below the minimum interspecific divergence (8.6%) previously reported within the genus *Scincella* (Bragin et al. 2025). The observed genetic divergence may reflect historical isolation associated with the complex topography of the Red River (Song Hong) Basin, combined with the limited dispersal ability of mountain *Scincella* species. Nonetheless, the degree of genetic separation does not appear sufficient to warrant species-level differentiation at this time.

## Discussion

Biodiversity surveys are fundamental for documenting species distributions and providing information for conservation priorities. The discovery of *Scincellafansipanensis* in Mt. Ailao, Yunnan Province, China, represents a notable range extension from the type locality in the Hoang Lien Son Range of northern Vietnam. Despite this geographic disjunction, specimens from both regions are morphologically and chromatically consistent, supporting their conspecific status. Minor differences, such as a higher number of gular and ventral scales and slightly shorter fore-limbs in the Chinese population, may reflect localised phenotypic plasticity or adaptation to different microhabitats and elevations. This record increases the number of *Scincella* species known from China to 15 and those recorded in Yunnan Province to five, suggesting that the region’s herpetofaunal diversity remains underestimated. The current distribution of *S.fansipanensis* across northern Vietnam and central Yunnan highlights the ecological continuity of the montane forests stretching along the south-eastern Himalayas and the northern Indochinese highlands.

Field observations suggest that *Scincellafansipanensis* is ecologically specialised for grassy, open-canopy habitats at high elevations, such as forest edges and canopy gaps, distinguishing it from many congeners that inhabit lower or more densely forested environments. The species appears to be sensitive to light availability and vegetation structure and is rarely encountered in heavily shaded primary forest. This microhabitat preference, while indicating specialisation, also raises potential conservation concerns, as forest edge habitats are often susceptible to anthropogenic disturbance. Nevertheless, the species’ presence near forest-settlement interfaces may indicate some tolerance to moderate habitat modification. Geographically, the Ailao Mountain Range in central Yunnan extends northwest-southeast towards the China-Vietnam border, while the Hoang Lien Son Range lies further east, with its northernmost ridges extending into southern Yunnan. Although adjacent, the two mountain systems are separated by the complex topography of the Red River (Song Hong) valley, which likely acts as a barrier to gene flow and may promote genetic divergence. Notably, the elevation of the new records (2,480 m) is consistent with that of the type locality (2,282–2,366 m), further supporting the ecological similarity of the two sites.

Additional surveys in the intervening regions are needed to determine whether the observed disjunct distribution reflects true biogeographic separation or a sampling gap. Molecular analyses would be particularly valuable to confirm the taxonomic identity of the Chinese population and assess potential genetic structure or divergence. A clearer understanding of population connectivity and the species’ full distribution is essential for informed conservation planning.

## Supplementary Material

XML Treatment for Scincella fansipanensis

## Figures and Tables

**Table 1.** GenBank accession numbers, localities and voucher information for all specimens used in this study.

**No.**	**Species name**	**Locality**	**Voucher NO.**	***12S***	***16S***	***CO1***	**References**
1	*Scincellafansipanensis*	Mt. Ailao, Jingdong, Yunnan, China	QHU R2025004	PV747499	PV747739	PV747750	This study
2	*Scincellafansipanensis*	Mt. Ailao, Jingdong, Yunnan, China	QHU R2025005	PV747500	PV747740	PV747751	This study
3	*Scincellafansipanensis*	Mt. Ailao, Jingdong, Yunnan, China	QHU R2025006	PV747501	PV747741	PV747752	This study
4	*Scincellafansipanensis*	Mt. Ailao, Jingdong, Yunnan, China	QHU R2025007	PV747502	PV747742	PV747753	This study
5	*Scincellafansipanensis*	Mt. Fansipan, Lao Cai, Vietnam	IEBR R.5185	–	–	LC846671	Okabe et al. (2024)
6	*Scincellafansipanensis*	Mt. Fansipan, Lao Cai, Vietnam	IEBR R.5187	–	–	LC846672	Okabe et al. (2024)
7	*Scincellafansipanensis*	Mt. Fansipan, Lao Cai, Vietnam	IEBR R.5188	–	–	LC846673	Okabe et al. (2024)
8	*Scincellafansipanensis*	Mt. Fansipan, Lao Cai, Vietnam	IEBR R.5189	–	–	LC846674	Okabe et al. (2024)
9	*Scincellafansipanensis*	Mt. Fansipan, Lao Cai, Vietnam	IEBR R.5192	–	–	LC846675	Okabe et al. (2024)
10	*S.alia*	Mt. Tay Con Linh, Ha Giang, Vietnam	VRTC NAP14081	–	–	PV085567	Bragin et al. (2025)
11	*S.alia*	Mt. Tay Con Linh, Ha Giang, Vietnam	ZMMU Re-18153	PV088911	PV088913	PV085569	Bragin et al. (2025)
12	*S.assata*	Finca El Milagro, Santa Ana, El Salvador	KU 289795	JF497946	JF498074	–	Linkem et al. (2011)
13	*S.assata*	Canton El Volcan, San Miguel, El Salvador	KU 291286	–	JF498075	–	Linkem et al. (2011)
14	*S.badenensis*	Mt. Ba Den, Tay Ninh, Vietnam	ITBCZ 5966	–	–	MK990602	Nguyen et al. (2019)
15	*S.badenensis*	Mt. Ba Den, Tay Ninh, Vietnam	ITBCZ 5993	–	–	MK990603	Nguyen et al. (2019)
16	*S.baraensis*	Mt. Ba Ra, Binh Phuoc, Vietnam	ITBCZ 6534	–	–	MT742256	Nguyen et al. (2020)
17	*S.baraensis*	Mt. Ba Ra, Binh Phuoc, Vietnam	ITBCZ 6536	–	–	MT742258	Nguyen et al. (2020)
18	*S.boettgeri*	Yaeyama Group, Southern Ryukyus, Japan	KUZ R68001	–	–	LC630768	Koizumi et al. (2022)
19	*S.boettgeri*	Yaeyama Group, Southern Ryukyus, Japan	KUZ R68008	–	–	LC630770	Koizumi et al. (2022)
20	*S.chengduensis*	Dayi, Sichuan, China	CIB 107637	PQ466924	PQ466921	PQ467109	Jia et al. (2025)
21	*S.chengduensis*	Chongzhou, Sichuan, China	CIB 118786	PQ466923	PQ466920	PQ467108	Jia et al. (2025)
22	*S.cherriei*	Montes Azules Biosphere Reserve, Chiapas, Mexico	RCMX 219	–	MW265931	–	Castiglia et al. (2020)
23	*S.cherriei*	Montes Azules Biosphere Reserve, Chiapas, Mexico	RCMX 235	–	MW265932	–	Castiglia et al. (2020)
24	*S.devorator*	Ba Vi NP, Ha Noi, Vietnam	ZMMU NAP07169	PV088910	PV088912	PV085573	Bragin et al. (2025)
25	S.cf.doriae	Bidoup-Nui Ba NP, Lam Dong, Vietnam	ZMMU R‑13268‑00412	–	–	MH119616	Unpublished
26	S.cf.doriae	Bidoup-Nui Ba NP, Lam Dong, Vietnam	ZMMU R‑13268‑01062	–	–	MH119617	Unpublished
27	*S.dunan*	Yonagunijima Is, Southern Ryukyus, Japan	KUZ R65170	–	–	LC630778	Koizumi et al. (2022)
28	*S.dunan*	Yonagunijima Is, Southern Ryukyus, Japan	KUZ R67027	–	–	LC630779	Koizumi et al. (2022)
29	*S.formosensis*	Taiwan, China	KUZ R37515	–	–	LC630789	Koizumi et al. (2022)
30	*S.formosensis*	Taiwan, China	KUZ R37516	–	–	LC630790	Koizumi et al. (2022)
31	*S.gemmingeri*	Teocelo, Mexico	LSUMZ H-14810	AY308294	AY308445	–	–
32	*S.huanrenensis*	Pyeongchang‑gun, Gangwon‑do, Korea	G390SH	KU507306	KU507306	KU507306	Park et al. (2016)
33	*S.huanrenensis*	Pyeongchang‑gun, Gangwon‑do, Korea	–	NC030779	NC030779	NC030779	Park et al. (2016)
34	*S.lateralis*	Texas, USA	DCC 2842	HM852476	HM852503	–	Reeder and Reichert (2011)
35	*S.lateralis*	Texas, USA	KU 289460	JF497948	JF498077	–	Linkem et al. (2011)
36	*S.liangshanensis*	Meigu, Sichuan, China	CIB 119513	PP826317	PP826315	PP824806	Jia et al. (2024)
37	*S.liangshanensis*	Meigu, Sichuan, China	CIB 119514	PP826318	PP826314	PP824804	Jia et al. (2024)
38	*S.melanosticta*	Kon Chu Rang NR, Gia Lai, Vietnam	ZMMU NAP‑05519	–	–	MH119621	Neang et al. (2018)
39	*S.melanosticta*	Kon Chu Rang NR, Gia Lai, Vietnam	ZMMU NAP‑06376	–	–	MH119622	Neang et al. (2018)
40	*S.modesta*	Ningbo, Zhejiang, China	CIB 121415	PP819198	PP819195	PP819217	Jia et al. (2024)
41	*S.modesta*	Ningbo, Zhejiang, China	WYF 11520	PP819197	–	PP819215	Jia et al. (2024)
42	*S.monticola*	Shangri‑La, Yunnan, China	DL‑YNJC2020824	OP955952	OP955962	–	Jia et al. (2023)
43	*S.nigrofasciata*	Keo Seima WS, Mondulkiri, Cambodia	CBC02545	–	–	MH119613	Neang et al. (2018)
44	*S.nigrofasciata*	Dak Nong UNESCO Global Geopark, Vietnam	ITBCZ 11028	–	–	PQ634873	Nguyen et al. (2024)
45	*S.potanini*	Kangding, Sichuan, China	DL KD202109071	OP942203	OP935937	OP942210	Jia et al. (2023)
46	*S.potanini*	Kangding, Sichuan, China	DL KD202109072	OP942208	OP935987	OP942209	Jia et al. (2023)
47	*S.reevesii*	Zhaoqing, Guangdong, China	NB 2017030715	NC054206	NC054206	NC054206	Zhong et al. (2021)
48	*S.reevesii*	Zhaoqing, Guangdong, China	–	MN832615	MN832615	MN832615	Zhong et al. (2021)
49	S.cf.rufocaudata	Ke Go NR, Ha Tinh, Vietnam	ZFMK 76238	–	HM773216	–	Nguyen et al. (2011)
50	S.cf.rufocaudata	Ke Go NR, Ha Tinh, Vietnam	ZFMK 76239	–	HM773217	–	Nguyen et al. (2011)
51	*S.rupicola*	Thailand	KUZ 40458	AB057388	AB057403	–	Honda et al. (2003)
52	*S.vandenburghi*	Tsushima Island, Japan	KUZ R66394	–	–	LC507695	Koizumi et al. (2021)
53	*S.vandenburghi*	Yeongwol‑gun, Korea	G389SV	KU646826	KU646826	KU646826	Park et al. (2016)
54	*S.wangyuezhaoi*	Wenchuan, Sichuan, China	CIB 87246	OP942191	OP941172	OQ402205	Jia et al. (2023)
55	*S.wangyuezhaoi*	Lixian, Sichuan, China	CIB 119510	OP942192	OP941174	–	Jia et al. (2023)
	Out group						
56	*Sphenomorphuscryptotis*	Shangsi, Guangxi, China	CIB 119027	OP942206	OP942190	OP942215	Jia et al. (2023)

**Table 2.** Main morphological characteristics of *Scincellafansipanensis* specimens from Yunnan Province, China. All measurements are in mm, the abbreviations of morphological characters are defined in the Materials and Methods section.

**Voucher Number**	**QHU R202500 4**	**QHU R202500 5**	**QHU R202500 6**	**QHU R2025007**
**Sex**	Male	Male	Juvenile	Juvenile
**Original tail**	No	No	Yes	No
**SVL**	50.7	51.5	33.5	30.1
**TAL**	–	–	59.7	–
**TAL/SVL**	–	–	1.78	–
**AGD**	29.8	30.1	19.9	16.5
**AGD/SVL**	0.59	0.58	0.59	0.55
**HL 1**	8.96	9.03	6.62	5.72
**HL2**	7.40	7.82	5.75	5.18
**HW**	5.16	5.29	4.13	4.00
**HH**	4.69	4.49	3.03	2.83
**ED**	2.15	2.12	1.71	1.52
**ESD**	2.28	2.39	1.81	1.57
**EN**	1.93	1.71	1.54	1.06
**PDD**	0.89	0.84	0.58	0.60
**EL**	1.58	1.45	1.04	1.16
**EL/PDD**	1.78	1.73	1.79	1.93
**FLL**	8.90	8.46	6.13	5.39
**FLL/SVL**	0.18	0.16	0.18	0.18
**HLL**	12.02	11.36	8.80	7.02
**HLL/SVL**	0.24	0.22	0.26	0.23
**F4S**	1.95	1.92	1.42	1.44
**T4S**	3.57	2.93	2.07	2.02
**PF**	2, separated	2, separated	2, separated	2, separated
**FrP**	in contact	in contact	in contact	in contact
**P**	in contact	in contact	in contact	in contact
**SO**	4	4	4	4
**SCI**	6/5	6/6	6/6	6/6
**Lor**	2	2	2	2
**TEMP**	1+2/1+2	1+2/1+2	1+2/1+2	1+2/1+2
**SL**	7	7	7	7
**IL**	6	6	6	6
**Chin shields (pair)**	3	3	3	3
**NU**	3/4	2/2	3/4	2/2
**MBSR**	22	22	22	24
**PVSR**	65	64	71	71
**DBR**	1/2+4+1/2	1/2+4+1/2	1/2+4+1/2	1/2+4+1/2
**GS**	22	19	22	21
**VS**	48	53	52	49
**GS+VS**	70	72	74	70
**F4S**	8	8	7	8
**T4S**	10	12	11	11
**SRB**	1.5–2	1.5–2	2	1.5–2
**UMLLS**	straight	straight	straight	straight
**VDM**	spots	spots	absent	spots
**Limbs adpressed**	No	No	No	No

**Table 3.** Uncorrected *p*-distances of the *CO1* gene amongst species of the *Scincellapotanini*-*S.monticola* complex included in the phylogenetic analyses of this study.

**No.**	**Species**	1–4	5–9	10–11	12–13	14	15–16	17–18	19–20
1–4	*S.fansipanensis* (China)	0.0–2.0							
5–9	*S.fansipanensis* (Vietnam)	6.5–7.3	0.0–0.2						
10–11	*S.alia*	18.9–19.7	18.0–19.1	0.8					
12–13	*S.chengduensis*	19.2–20.1	18.4–19.3	16.8–17.4	0.3				
14	*S.devorator*	16.2–17.4	16.1–16.3	20.6–20.9	17.9–18.3	–			
15–16	*S.liangshanensis*	12.8–13.8	14.2–14.7	19.3–19.8	18.0–19.0	17.9–18.3	1.2		
17–18	*S.potanini*	16.5–17.0	16.9–17.4	20.5–22.0	19.5–20.2	16.9–17.4	16.2–16.9	0.3	
19–20	*S.truongi*	18.9–19.4	19.4–19.6	20.0–20.1	19.0–19.4	21.4	21.8–22.8	22.6–23.2	0.0
